# Relapse prevention with cariprazine in patients with early-stage schizophrenia

**DOI:** 10.1192/j.eurpsy.2024.606

**Published:** 2024-08-27

**Authors:** C. U. Correll, Z. B. Dombi, P. L. Herman, Á. Barabássy

**Affiliations:** ^1^The Donald and Barbara Zucker School of Medicine at Hofstra/Northwell, New York, United States; ^2^Charité – Universitätsmedizin, Berlin, Germany; ^3^Global Medical Division, Gedeon Richter Plc., Budapest, Hungary

## Abstract

**Introduction:**

Relapse is defined as the return of psychotic symptoms after a period of improvement/stability. Relapse is often associated with the disruptive re-hospitalization of patients. Importantly, relapse history is a strong predictor of subsequent relapses and poorer outcomes. Therefore, relapse prevention in the beginning of the disorder is especially important. Cariprazine, a novel D_3_-D_2_ partial agonist, has been effective in preventing relapse compared to placebo in stabilized patients with schizophrenia.

**Objectives:**

To present the efficacy of cariprazine in preventing relapse in patients with early-stage schizophrenia.

**Methods:**

Post-hoc analysis of data from a ˜96 weeks, multicentre, randomized, double-blind, placebo-controlled, parallel-group study in adults with schizophrenia. The study was composed of two parts: a 20-week open-label treatment phase and a double-blind treatment phase up to 72 weeks. During the open-label phase, patients were stabilized with cariprazine 3.0-9.0 mg/day. Then, they were randomized to continue cariprazine (fixed dosing: 3.0, 6.0, or 9.0 mg/day) or receive placebo. Relapse was defined as a deterioration of symptom scores as measured by the Positive Negative Syndrome Scale (PANSS), admission to a psychiatric hospital, exhibiting aggressive behaviour, or risk of suicide. In the present analysis, patients with a schizophrenia diagnosis history of 0-5 years were defined as early-stage patients. Baseline characteristics, and risk ratios (after the double-blind phase) with number-needed-to-treat (NNT) were calculated.

**Results:**

Of 200 patients, 71 (35.5%) met the early-stage criteria: 32 patients in the cariprazine (CAR) and 39 in the placebo (PBO) arm. The mean age was 31.6 years in both groups with an average illness duration of 2.51+/-1.03 years in the CAR and 2.75+/-1.24 years in the PBO arm. 47% of patients in the CAR arm and 77% in the PBO arm were men. The average number of previous hospitalisations was comparable in the two groups (CAR: 2.3; PBO: 2.6), as was the severity of illness: mean PANSS Total score: 89.2 (CAR), 90.4 (PBO). Patients in both groups were highly compliant (pill-count: CAR: 98.2%; PBO: 99.5%). The main reported adverse effects were headache (CAR: 11.3%, PBO: 7.0%), insomnia (CAR: 5.6%, PBO: 4.2%), and increased triglycerides (CAR: 5.6%, PBO: 1.4%), discontinuation due to adverse event was 3.1% in the CAR and 2.6% in the PBO group. Altogether, 9.4% of patients relapsed in the cariprazine group compared to 48.7% on placebo (risk ratio=0.19 (95% confidence interval (CI): 6.3-59.2%, p=0.0041;NNT: 2.5 (95%CI: 1.7-5.1).

**Conclusions:**

In this post-hoc analysis of patients within the first five years of schizophrenia, the relative risk of relapse was 81% reduced with cariprazine with prevention of one additional relapse after each third patient exposed to cariprazine vs placebo. Cariprazine seems to be a good treatment option for early-stage patients for preventing relapse.

**Disclosure of Interest:**

C. Correll Consultant of: AbbVie, Acadia, Alkermes, Allergan, Angelini, Aristo, Biogen, Boehringer-Ingelheim, Cardio Diagnostics, Cerevel, CNX Therapeutics, Compass Pathways, Darnitsa, Denovo, Gedeon Richter, Hikma, Holmusk, IntraCellularTherapies, Jamjoom Pharma, Janssen/J&J, Karuna, LB Pharma, Lundbeck, MedAvante-ProPhase, MedInCell, Merck, Mindpax, Mitsubishi Tanabe Pharma, Mylan, Neurocrine, Neurelis, Newron, Noven, Novo Nordisk, Otsuka, Pharmabrain, PPD Biotech, Recordati, Relmada, Reviva, Rovi, Sage, Seqirus, SK Life Science, Sumitomo Pharma America, Sunovion, Sun Pharma, Supernus, Takeda, Teva, Tolmar, Vertex, and Viatris., Z. Dombi Employee of: Gedeon Richter Plc., P. Herman Employee of: Gedeon Richter Plc., Á. Barabássy Employee of: Gedeon Richter Plc.

